# An observational study on salivary conductivity for fluid status assessment and clinical relevance in acute ischemic stroke during intravenous fluid hydration

**DOI:** 10.1038/s41598-023-49957-7

**Published:** 2023-12-18

**Authors:** Chun-Hao Chen, An-Ting Lee, Jen-Tsung Yang, Yuan-Hsiung Tsai, Leng-Chieh Lin, Yen-Chu Huang

**Affiliations:** 1 Department of Orthopedic, Chang Gung Memorial Hospital Chiayi Branch, Puzi, Taiwan; 2 Department of Anesthesiology, Chang Gung Memorial Hospital Chiayi Branch, Puzi, Taiwan; 3 Department of Neurosurgery, Chang Gung Memorial Hospital Chiayi Branch, Puzi, Taiwan; 4 Present Address: Department of Radiology, Chang Gung Memorial Hospital Chiayi Branch, Puzi, Taiwan; 5 Department of Emergency Medicine, Chang Gung Memorial Hospital, Chiayi Branch, Puzi, Taiwan; 6 Department of Neurology, Chang Gung Memorial Hospital, Chiayi Branch, No. 6 West Chia-Pu Road, Putz, Chiayi County Taiwan; 7 College of Medicine, Chang Gung University, Taoyuan, Taiwan

**Keywords:** Neuroscience, Diseases, Neurology

## Abstract

The body fluid status in acute stroke is a crucial determinant in early stroke recovery but a real-time method to monitor body fluid status is not available. This study aims to evaluate the relationship between salivary conductivity and body fluid status during the period of intravenous fluid hydration. Between June 2020 to August 2022, patients presenting with clinical signs of stroke at the emergency department were enrolled. Salivary conductivities were measured before and 3 h after intravenous hydration. Patients were considered responsive if their salivary conductivities at 3 h decreased by more than 20% compared to their baseline values. Stroke severity was assessed using the National Institutes of Health Stroke Scale, and early neurological improvement was defined as a decrease of ≥ 2 points within 72 h of admission. Among 108 recruited patients, there were 35 of stroke mimics, 6 of transient ischemic attack and 67 of acute ischemic stroke. Salivary conductivity was significantly decreased after hydration in all patients (9008 versus 8118 µs/cm, *p* = 0.030). Among patients with acute ischemic stroke, the responsive group, showed a higher rate of early neurological improvement within 3 days compared to the non-responsive group (37% versus 10%, *p* = 0.009). In a multivariate logistic regression model, a decrease in salivary conductivity of 20% or more was found to be an independent factor associated with early neurological improvement (odds ratio 5.42, 95% confidence interval 1.31–22.5, *p* = 0.020). Real-time salivary conductivity might be a potential indicator of hydration status of the patient with acute ischemic stroke.

## Introduction

Stroke is a leading cause of death and complex disability worldwide, which can lead to serious financial issues for patients and their families^1,2^. Appropriate treatment of ischemic stroke is essential for reducing mortality and morbidity^3^. Restoration of the hemodynamic compromise and maintenance of the collateral flow are critical in the acute stage of ischemic stroke^4^. The hydration status at the time of stroke has been recognized as an important determinant in early stroke recovery^5^. Reduced intravascular volume upon ischemic stroke has been associated with in-hospital complications, disability, and mortality^6^. Dehydration is common at the time of stroke due to underlying diseases and swallowing dysfunction^7^, and may lead to a reduction of cerebral perfusion and reduce the possibility of early stroke recovery^8^. Current guidelines recommend intravenous fluid supplementation for all patients with ischemic stroke especially when they are dehydrated^9^.

However, the definition of dehydration is imprecise, and recommendations for hydration therapy are scarce^10^. In addition, there is no gold standard for the diagnosis of ‘dehydration’ or “inadequate fluid status” in acute stroke patients^11,12^. Despite availability of bedside assessment of hydration status, its application in acute stroke treatment remains uncertain. Clinically, several blood and urine biomarkers are used to evaluate dehydration, including serum osmolality, urine-specific gravity and osmolality, and serum blood urine nitrogen to creatine ratio^11^. Although these tests are widely performed, the minimally invasive procedures can become burdensome, especially when repeated within a short period of time. Although urine specimens are non-invasive to collect, urine voiding may not be readily available, especially in dehydrated patient. Moreover, these biomarkers may not fully represent the real-time fluid status and cerebral perfusion^13,14^. The diagnosis of dehydration, or more accurately, a volume-contracted state, at the time of stroke is challenging as there are currently no consensus diagnostic criteria.

To address these limitations, we developed a portable device for measuring salivary conductivity, which is highly associated with fluid status^15,16^. This allows for a non-invasive and real-time evaluation of fluid status, and the intensity of hydration treatment may be adjusted in real-time to improve clinical outcomes after acute stroke. Therefore, the purpose of this pilot study was to evaluate the relationship between fluid status and the early clinical response using real-time salivary conductivity in patients with acute ischemic stroke.

## Materials and methods

### Ethical statement

This longitudinal observational study was conducted in compliance with the guiding principles of the Declaration of Helsinki and was approved by the Medical Ethics Committee of Chang Gung Memorial Hospital (202001388A3C501). Prior to the study, all subjects provided written informed consent.

### Study subjects

**Figure 1:**
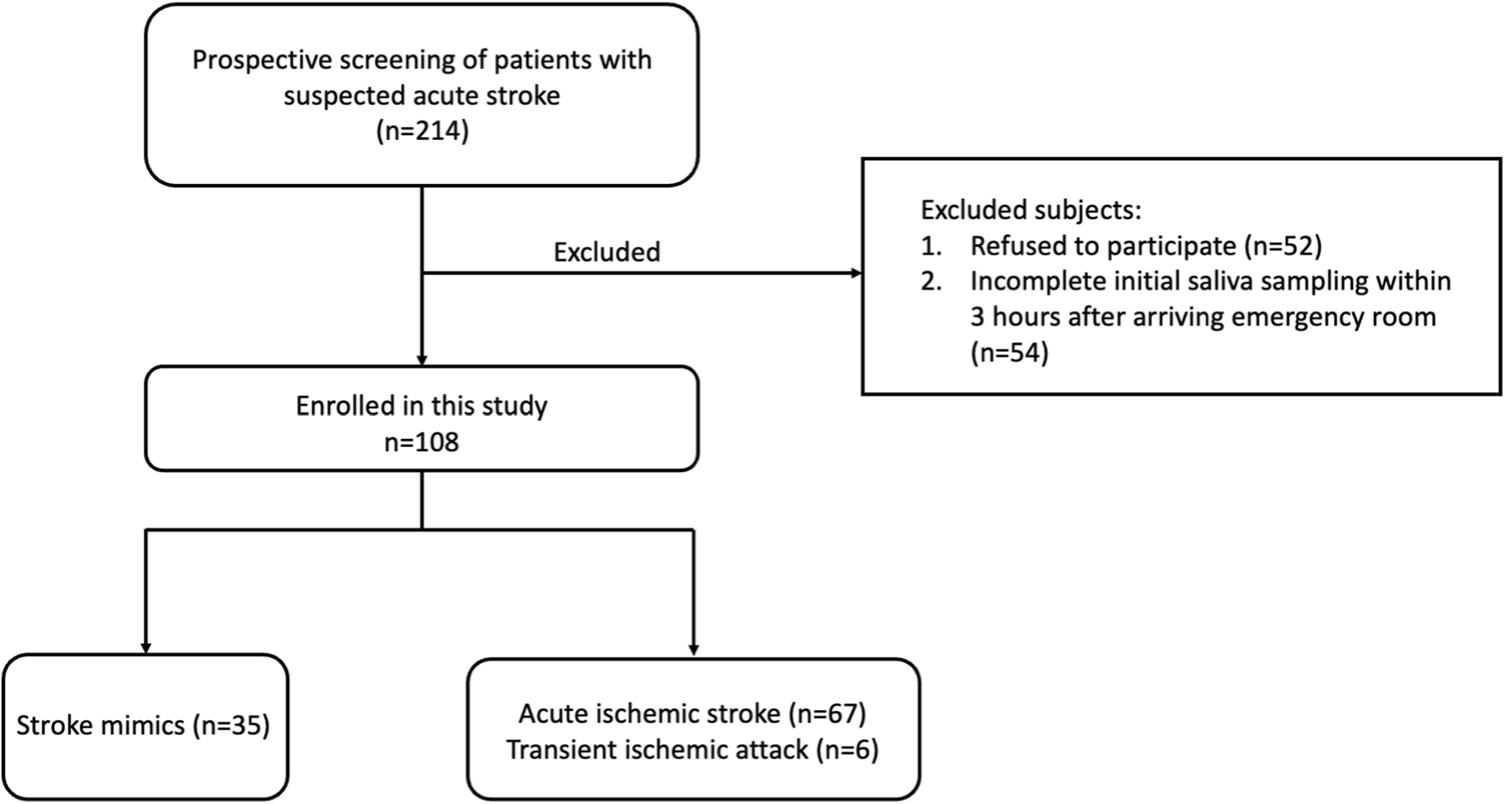
Flow chart. Diagram shows the enrolment and status of patients.

From September 2020 to May 2021, we screened 214 patients who presented with stroke symptoms at the emergency department of Chang Gung Memorial Hospital in Chiayi, Taiwan. Out of these, 106 patients were excluded from the study as 52 patients refused to participate, and 54 patients did not complete saliva sampling before hydration upon arriving the ER. Among the remaining 108 eligible patients, 67 patients were diagnosed with acute ischemic stroke, as evidenced by visible ischemic lesions on the subsequent MRI scans, while the other 35 patients were classified as stroke mimics and 6 patients were classified as transient ischemic attack (TIA) (Fig. 1).

### Experimental procedures

We explained the experimental design to all patients at the beginning of the study, which included instructions on the collection of saliva, as well as hematological and biochemical examinations. The hydration protocol involved administering a consistent amount of fluids to the patients upon their arrival in the emergency department, at a continuous infusion rate of 80 cm^3^ per hour. Hydration was provided to all patients unless contraindications were present, such as heart failure or end-stage renal artery disease. Data collection took place at three distinct time points: at the onset of the study, after 3-h period of hydration with normal saline, and 3 days later. There were no restrictions placed on their physical activity following admission.

### Salivary collection and analysis

**Figure 2:**
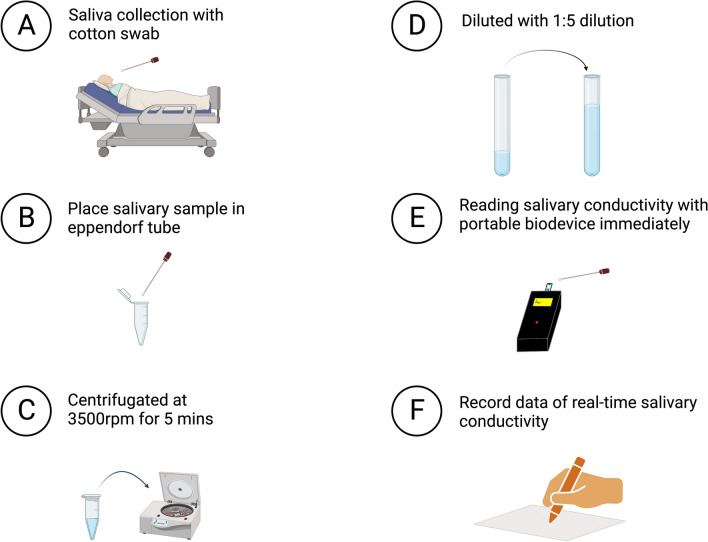
Salivary collection and analysis protocol. Salivary conductivity is measured using a printed-circuit board. (**A**) Collect saliva smoothly with a cotton swab. (**B**) Place salivary sample in an Eppendorf tube after collection. (**C**) Centrifuge the salivary sample at 3500 rpm for 5 min. (**D**) Dilute the salivary sample fivefold with ultrapure water after centrifugation. (**E**) Analyze salivary conductivity using a developed portable bio-device equipped with a disposable printed-circuit board electrode. (**F**) Record real-time data of salivary conductivity immediately.

We collected sublingual saliva from the floor of the mouth using a cotton swab and placed it into a 1.5 mL Eppendorf tube. The salivary sample was centrifugated at 3500 rpm for 5 min and then diluted fivefold with ultrapure water. Salivary conductivity was analyzed using a developed portable monitor equipped with a disposable printed-circuit board electrode. Real-time data on salivary conductivity were immediately recorded (Fig. 2).

### Clinical evaluation of severity of stroke and clinical outcome

In this study, stroke severity was assessed using the National Institutes of Health Stroke Scale (NIHSS), and early neurological improvement (ENI) was defined as a decrease of ≥ 2 points on the NIHSS within 72 h of admission. However, patients experiencing fluctuating neurological deficits due to concurrent infections, medications or poorly managed blood sugar levels were not included. The modified Rankin Scale was evaluated at 3 months post-stroke. During hydration therapy, patients were categorized as responsive if their salivary conductivities at 3 h showed a decrease of over 20% from the baseline values.

### Statistical analysis

Continuous variables are presented as the mean ± standard deviation, while categorical variables are presented as numbers and percentages. The Kolmogorov–Smirnov test was used to examine the normalization of continuous variables. Independent-sample *t*-test or Mann–Whitney *U* test was used to compare quantitative variables between the two groups, as appropriate, whereas chi-squared or Fisher’s exact test were used for categorical variables. Univariate binary logistic regression analysis was performed to compare the frequency of potential factors associated with clinical outcomes. To control for confounding factors, a Forward conditional multivariate binary logistic regression analysis was conducted on variables that showed significance on univariate analysis. The criterion for significance to reject the null hypothesis was a 95% confidence interval. IBM SPSS Statistics Version 25 for MAC (IBM corporation, Armonk, NY, USA) as used for statistical analyses.

### Ethics approval and consent to participate

The protocols of these studies were approved by the Institutional Review Board of Chang Gung Memorial Hospital (202001388A3C501). All procedures performed in studies involving human participants were in accordance with the ethical standards of the institutional and/or national research committee and with the 1964 Helsinki declaration and its later amendments or comparable ethical standards. Informed consent was obtained from all individual participants.

## Results

**Figure 3:**
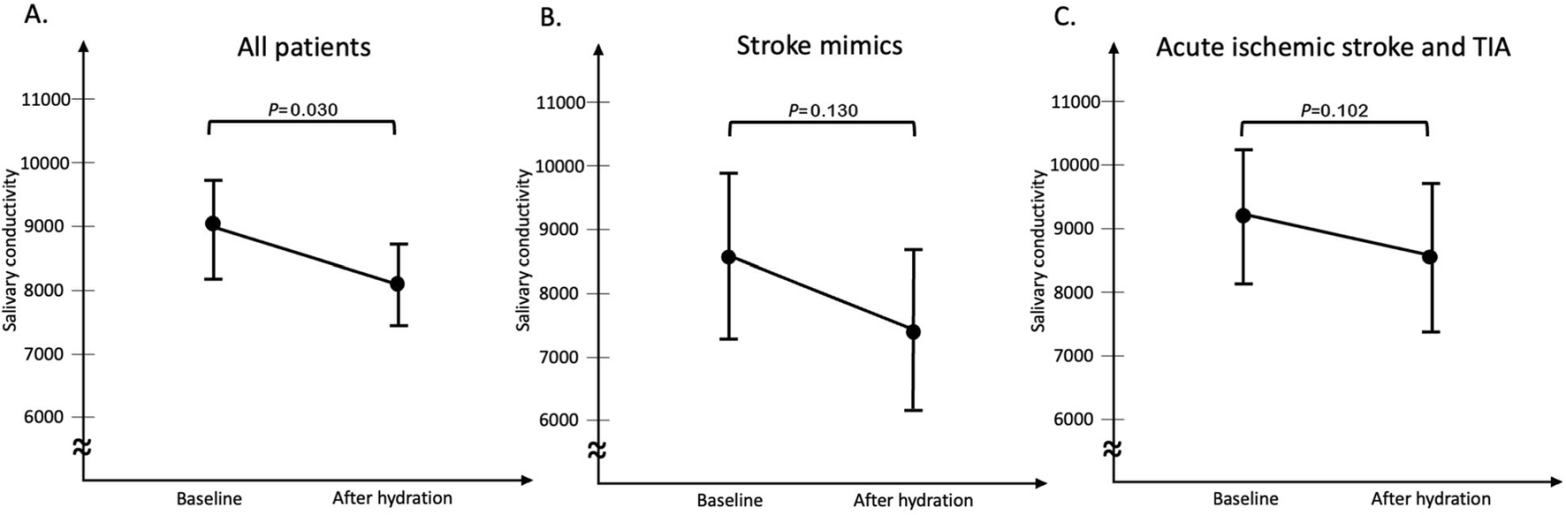
Salivary conductivity changes following hydration therapy in all patients (**A**) and different groups of stroke mimics (**B**) and ischemic stroke (**C**). Note: The black dots and error bars represent the mean and standard error of salivary conductivity.

In this study, 108 patients with clinically suspected stroke in the emergency department were recruited, including 35 of stroke mimics, 6 of TIA and 67 of acute ischemic stroke. We analyzed demographic data from two groups: patients with stroke mimics and those with acute ischemic stroke and TIA (Table 1). The mean age of the patients was 69.2 ± 12.6 years. Patients with acute ischemic stroke or TIA were older (71.1 vs. 66.0; *p* = 0.044) compared to those with stroke mimics. There was no significant difference between the two groups in salivary conductivity at arrival and underlying diseases, including hypertension, diabetes mellitus, hyperlipidemia, atrial fibrillation, coronary artery disease, and chronic kidney disease. Laboratory data of serum glucose and estimated glomerular filtration rate were not significantly different between two groups. The salivary conductivities measured before and 3 h after hydration therapy were higher in patients with acute ischemic stroke and TIA than in those of stroke mimics, but the difference did not reach a statistical significance (9171 vs. 8666 µs/cm, p = 0.535; 8404 vs. 7512 µs/cm, p = 0.330). Compared to the baseline status, salivary conductivity decreased significantly after hydration (9008 vs. 8118 µs/cm, p = 0.030) (Fig. 3). The decreasing trends were observed in both subgroups of stroke mimics (8666 vs. 7512 µs/cm, *p* = 0.130) and acute ischemic stroke/TIA (9171 versus 8404 µs/cm, *p* = 0.102) (Fig. 3).

**Table 1:** Baseline characteristics of participants stratified by groups.

	All	Stroke mimics	Ischemic stroke/TIA	*p* value
Number	108	35	73	
Age, years	69.2 ± 12.6	66.0 ± 13.8	71.1 ± 11.7	0.044*
Sex, female gender	39 (36%)	12 (34%)	27 (37%)	0.784
Hypertension	66 (61%)	22 (63%)	44 (60%)	0.797
Diabetes mellitus	37 (34%)	11 (31%)	26 (36%)	0.668
Hyperlipidemia	27 (25%)	10 (29%)	17 (23%)	0.553
Atrial fibrillation	11 (10%)	1 (3%)	10 (14%)	0.099
Coronary artery disease	4 (4%)	2 (6%)	2 (3%)	0.594
Old stroke history	19 (18%)	7 (20%)	12 (16%)	0.649
Chronic kidney disease	5 (5%)	3 (9%)	2 (3%)	0.326
Cigarette smoking	34 (31%)	9 (26%)	25 (34%)	0.372
Blood urea nitrogen, mg/dL	18.5 ± 12.7	20.3 ± 19.4	17.6 ± 7.5	0.436
Creatinine	1.0 ± 0.5	1.0 ± 0.6	1.0 ± 0.5	0.803
eGFR, mL/min/1.73 m^2^	77.1 ± 26.5	80.0 ± 28.6	75.8 ± 25.5	0.451
Serum glucose, mg/dL	138.7 ± 51.5	130.2 ± 41.0	142.6 ± 55.5	0.177
Sodium, mEq/L	137.6 ± 4.0	136.6 ± 5.7	138.1 ± 2.6	0.367
Blood osmolality, mOsm/kgH_2_O	296.8 ± 9.6	294.8 ± 14.6	297.7 ± 5.7	0.284
NIHSS at arrival	5.2 ± 4.5	N/A	5.2 ± 4.5	N/A
NIHSS at 3 days	4.6 ± 4.3	N/A	4.7 ± 4.3	N/A
Salivary conductivity at arrival, µs/cm	9008 ± 3938	8666 ± 4458	9171 ± 3685	0.535
Salivary conductivity at 3 h, µs/cm	8118 ± 3715	7512 ± 3108	8404 ± 3957	0.330
Salivary conductivity at 3 days, µs/cm	7262 ± 3390	7930 ± 3485	7193 ± 3410	0.584

Values are shown as the mean ± standard deviation. *eGFR* estimated glomerular filtration rate, *NIHSS* National Institutes of Health Stroke Scale, *TIA* transient ischemic attack.

*p < 0.05.

Table 2 shows a total of 67 patients with acute ischemic stroke, of whom 27 were classified as responsive to hydration therapy. Patients with serum osmolarity ≥ 300 mOsm/kgH_2_O had a higher salivary conductivity before hydration therapy than those with serum osmolarity < 300 mOsm/kgH_2_O (10,454 vs. 8426 µs/cm, p = 0.037). Compared to the non-responsive group, the responsive group had a higher salivary conductivity at arrival (10,738 vs. 8263 µs/cm; p = 0.007) and a higher rate of ENI within 3 days (37% vs. 10%, *p* = 0.009). The univariate logistic regression model showed that a decrease in salivary conductivity of ≥ 20%, a more severe NIHSS score and lower blood urea nitrogen were associated with ENI (Table 3). In the multivariate logistic regression model, a decrease in salivary conductivity of ≥ 20% and more severe NIHSS score were found to be an independent factor associated with ENI [odds ratio(OR) 5.42, 95% confidence interval (CI) 1.31–22.5, *p* = 0.020; OR 1.20, 95% CI 1.04–1.39, *p* = 0.016] (Table 3).

**Table 2:** Baseline characteristics of patients with or without salivary response after hydration.

	Responsive	Non-responsive	*p* value
Number	27	40	
Age, years	71.6 ± 11.5	72.4 ± 11.4	0.781
Sex, female gender	9 (33%)	15 (38%)	0.727
Hypertension	15 (56%)	27 (68%)	0.321
Diabetes mellitus	8 (30%)	17 (43%)	0.285
Hyperlipidemia	6 (22%)	8 (20%)	0.826
Atrial fibrillation	3 (11%)	7 (18%)	0.472
Coronary artery disease	1 (4%)	1 (3%)	1.000
Old stroke history	5 (19%)	6 (15%)	0.745
Chronic kidney disease	1 (4%)	1 (3%)	0.776
Cigarette smoking	8 (30%)	14 (35%)	0.043*
Blood urea nitrogen, mg/dL	17.9 ± 9.3	18.1 ± 6.5	0.898
Creatinine	1.0 ± 0.7	1.0 ± 0.3	0.227
eGFR, mL/min/1.73 m^2^	78.0 ± 22.3	71.0 ± 25.7	0.261
Serum glucose, mg/dL	149 ± 74.2	141.5 ± 44.9	0.553
Sodium, mEq/L	138.2 ± 2.4	137.8 ± 2.8	0.559
Blood osmolality, mOsm/kgH_2_O	298.0 ± 5.7	297.5 ± 5.9	0.731
NIHSS at arrival	6.1 ± 4.9	5.3 ± 4.3	0.410
NIHSS at 3 days	5.2 ± 2.3	5.0 ± 4.7	0.438
Early neurological improvement	10 (37%)	4 (10%)	0.009*
Salivary conductivity, at arrival, µs/cm	10,738 ± 3925	8263 ± 3257	0.007*
Salivary conductivity, at 3 h, µs/cm	7725 ± 3228	9243 ± 4355	0.132
Salivary conductivity, at 3 days, µs/cm	7561 ± 2880	7195 ± 4180	0.390
mRS at 3 months	2.2 ± 1.4	2.1 ± 1.4	0.705

Values are shown as the mean ± standard deviation. *eGFR* estimated glomerular filtration rate, *NIHSS* National Institutes of Health Stroke Scale.

*p < 0.05.

**Table 3:** Prediction factors for early neurological improvement in acute ischemic stroke.

	Univariate		Multivariate	
	Odds ratio (95% CI)	*p* value	Odds ratio (95% CI)	*p* value
Conductivity decreased ≥ 20%	5.15 (1.41–18.8)	0.013*	5.42 (1.31–22.5)	0.020*
NIHSS at arrival	1.19 (1.04–1.35)	0.009*	1.20 (1.04–1.39)	0.016*
Age, years	1.00 (0.95–1.05)	0.990		
Blood urea nitrogen, mg/dL	0.87 (0.77–0.99)	0.039*		
Creatinine	0.22 (0.02–2.39)	0.224		
eGFR, mL/min/1.73 m^2^	1.01 (0.99–1.04)	0.277		
Serum glucose, mg/dL	1.00 (0.98–1.01)	0.542		
Sodium, mEq/L	0.97 (0.78–1.21)	0.786		
Blood osmolality, mOsm/kgH_2_O	0.91 (0.82–1.02)	0.094		
Hypertension	1.30 (0.39–4.32)	0.666		
Diabetes mellitus	1.69 (0.47–6.12)	0.422		
Hyperlipidemia	4.33 (0.52–36.4)	0.177		
Cigarette	0.38 (0.11–1.32)	0.127		

*eGFR* estimated glomerular filtration rate, *NIHSS* National Institutes of Health Stroke Scale.

*p < 0.05.

## Discussion

Our pilot study revealed that salivary conductivity was correlated with fluid status in both ischemic stroke and stroke mimics, suggesting real-time changes of fluid status after intravenous hydration. The response of salivary conductivity was also related to ENI in patients of mild to moderate ischemic stroke. These preliminary results may shed light on real-time monitoring during fluid supplementation in acute ischemic stroke, and further studies are warrant to examine these findings.

Dehydration has been reported as an independent predictor of poor functional outcomes and high admission cost in acute ischemic stroke^17–19^. In a systematic research review by Bahouth et al., hydration status at the time of stroke was recognized as an important determinant in early stroke recovery. In this study, patients with higher osmolarity also had higher salivary conductivity upon arrival, and patients in the hydration-responsive group had higher initial salivary conductivity. These findings may suggest that salivary conductivity may reflect real-time fluid status and accurately detect dehydrated status. However, further studies with large sample sizes are needed to confirm these findings and determine their clinical usefulness.

Intravascular volume depletion is a common occurrence in acute stroke and may exacerbate cerebral blood flow^7^. Isotonic saline without dextrose is the preferred agent for intravascular fluid repletion and maintenance fluid therapy^20^. According Lin et al.^21^, dehydration is an independent predictor of early deterioration after acute ischemic stroke and rehydration can improve outcomes. Another study revealed that urine-specific gravity-based hydration might be a useful method for improving functional outcomes in non-hydrated patients with acute ischemic stroke at admission^22^. In a Phase III Trial, that the observed benefits of hydration therapy were prominent in patients with mild stroke, as supported by the findings from unpublished randomized controlled trial conducted by Lin et al.^23^. In our study, patients with acute ischemic stroke who responded to hydration therapy also had a higher chance of ENI, highlighting the importance of adequate hydration for patients with inadequate fluid status. However, patients without a decrease in salivary conductivity may also have inadequate hydration rather than non-dehydration initially.

In patients with acute ischemic stroke, we observed that salivary conductivity measured 3 h after fluid infusion was higher in non-responsive patients compared to the salivary conductivity recorded upon their arrival at the hospital. This could be due to the small amount of intravenous fluid supplementation, which may not be sufficient to correct dehydration caused by inadequate fluid intake related to stroke-related dysphagia or excessive loss of body fluids from the surface. Additionally, the delayed response of salivary conductivity to changes in bodily fluids might contribute to this phenomenon. Furthermore, with prolonged observation, a consistent decrease in salivary conductivity becomes evident, indicating that patients who receive consistent hydration exhibit a salivary conductivity response. Further studies are needed to clarify the association between fluid status and changes in salivary conductivity.

Many parameters have been used to evaluate fluid status in patients with various clinical conditions. Serum osmolality has been shown in several studies to accurately identify hydration state and is sensitive to changes in the dehydration state^10,24^. However, serum osmolality has a long turnover time and is invasive, making it difficult to reflect real-time hydration status^25,26^. Urine-specific gravity and urine osmolality are also very sensitive to changes in hydration status, but they lag behind body fluid turnover and are only moderately correlated with serum osmolality during acute dehydration^27–29^. Previous studies have shown that salivary conductivity is a rapid, real-time, and easy-to-administer screening tool for evaluating fluid status^15,16^. In addition, the saliva test method is simple and non-invasive, allowing for repeated measurements to monitor the fluid status during the period of hydration therapy in acute ischemic stroke.

There were several limitations in the present study. Firstly, the sample size was small, limiting the ability to fully explore the correlation between salivary conductivity and clinical significance in stroke patients. Due to the limited sample size, it is not possible in this study to further categorize the collected samples using TOAST stroke classification for additional meaningful classification and subsequent statistical analysis. Second, salivary conductivity may be influenced by age-related degeneration in the salivary glands^30,31^. Thirdly, there is a lack of reference ranges and identification of possible confounders for salivary conductivity. Fourthly, salivary conductivity indeed varies in response to changes in fluid status and other medical conditions. Various factors, such as age, impaired salivary functions due to conditions like Sjogren’s syndrome or post-radiation therapy status, serum glucose levels, medications, and endocrine disorders, can influence salivary secretion and conductivity. Impact of these confounding factors may potentially affect level of salivary conductivity. Despite these challenges, the study maintained its focus on observing variations in salivary conductivity following hydration and investigating their association with the clinical response. However, further well-designed prospective studies are warranted to confirm our findings.

## Conclusion

Real-time salivary conductivity might be a potential indicator of hydration status of the patients with acute ischemic stroke. Dehydration status may be associated with early neurologic improvement in patients with acute ischemic stroke. This portable monitor device can offer a novel and helpful parameter for the clinical evaluation of hydration status.

## Data Availability

The datasets during the current study are available from the corresponding author on reasonable request.
